# Mitochondrial Neurogastrointestinal Encephalomyopathy Caused by Thymidine Phosphorylase Enzyme Deficiency: From Pathogenesis to Emerging Therapeutic Options

**DOI:** 10.3389/fncel.2017.00031

**Published:** 2017-02-15

**Authors:** Rana Yadak, Peter Sillevis Smitt, Marike W. van Gisbergen, Niek P. van Til, Irenaeus F. M. de Coo

**Affiliations:** ^1^Department of Neurology, Erasmus University Medical CenterRotterdam, Netherlands; ^2^Department of Radiation Oncology (MaastRO-Lab), GROW – School for Oncology and Developmental Biology, Maastricht University Medical CentreMaastricht, Netherlands; ^3^Laboratory of Translational Immunology, University Medical Center UtrechtUtrecht, Netherlands

**Keywords:** mitochondrial neurogastrointestinal encephalomyopathy, MNGIE, thymidine phosphorylase, metabolic disease, HSCT, HSCGT, lentiviral vector

## Abstract

Mitochondrial neurogastrointestinal encephalomyopathy (MNGIE) is a progressive metabolic disorder caused by thymidine phosphorylase (TP) enzyme deficiency. The lack of TP results in systemic accumulation of deoxyribonucleosides thymidine (dThd) and deoxyuridine (dUrd). In these patients, clinical features include mental regression, ophthalmoplegia, and fatal gastrointestinal complications. The accumulation of nucleosides also causes imbalances in mitochondrial DNA (mtDNA) deoxyribonucleoside triphosphates (dNTPs), which may play a direct or indirect role in the mtDNA depletion/deletion abnormalities, although the exact underlying mechanism remains unknown. The available therapeutic approaches include dialysis and enzyme replacement therapy, both can only transiently reverse the biochemical imbalance. Allogeneic hematopoietic stem cell transplantation is shown to be able to restore normal enzyme activity and improve clinical manifestations in MNGIE patients. However, transplant related complications and disease progression result in a high mortality rate. New therapeutic approaches, such as adeno-associated viral vector and hematopoietic stem cell gene therapy have been tested in *Tymp^-/-^Upp1^-/-^* mice, a murine model for MNGIE. This review provides background information on disease manifestations of MNGIE with a focus on current management and treatment options. It also outlines the pre-clinical approaches toward future treatment of the disease.

## Introduction

Mitochondrial diseases represent a genetically and clinically heterogeneous group of disorders caused by mutations in mitochondrial DNA (mtDNA), that affect synthesis and function of mitochondrial proteins, such as tRNA (in MELAS disease) and ND1, 4, 6 (responsible for the majority of cases in LHON disease) (DiMauro, 2004). Another group is caused by mutations in nuclear DNA (nDNA) that lead to defects in nuclear encoded mitochondrial proteins. Part of these proteins exert their effect on mtDNA maintenance, thus known as nuclear-mitochondrial communication disorders. A subtype of the latter is MDS; a group of mainly autosomal recessive disorders caused by defects in nuclear genes involved in mtDNA replication (e.g., *POLG* and *PEO1* causing hepatocerebal MDS), or genes crucial for maintenance of mtDNA including *TK2* (responsible for myopathic MDS*), RRM2B* (encephalomypathic MDS) and thymidine phosphorylase (*TYMP*) gene mutations associated with MNGIE (El-Hattab and Scaglia, 2013). MNGIE, initially described in Okamura et al. (1976) is a fatal rare inherited metabolic disorder without genetic or ethnic predisposition (Gamez et al., 2005). The estimated rate of occurrence is 1–9:1,000,000^1^, and as of 2011 fewer than 200 cases have been described in the medical literature (Halter et al., 2011). Due to its variable clinical presentations, MNGIE can be easily overlooked or misdiagnosed as Crohn’s disease, psychiatric disorder, anorexia nervosa, or myasthenia gravis (Rickards et al., 1994; Teitelbaum et al., 2002; Marti et al., 2004).

## Genetic Defects, Clinical Manifestations, and Diagnosis

### Genetic Defects

Mitochondrial neurogastrointestinal encephalomyopathy is an autosomal recessive inherited disease that is caused by mutations in the nuclear gene *TYMP* (previously known as *ECGF1*). *TYMP* codes for the TP enzyme (EC 2.4.2.4.) and is located on chromosome 22q13.33 (Stenman et al., 1992). TP is a cytoplasmic enzyme expressed in most human tissues, including gastrointestinal tract, central and peripheral nervous system, spleen, liver, bladder, leukocytes and in platelets which account for most of the TP activity in human blood (Fukami and Salganicoff, 1973; Shaw et al., 1988). In contrast, TP is present at low levels in muscles and is lacking in kidney, aorta and fat tissues (Fox et al., 1995; Valentino et al., 2007). MNGIE is caused by a variety of pathogenic homozygous or compound heterozygous mutations in the exons or flanking regions of the *TYMP* gene. Various mutations are reported to date (Stenson et al., 2014) including deletions, single nucleotide insertions (Nishino et al., 1999), splice site (Kocaefe et al., 2003; Szigeti et al., 2004b) and frameshift mutations (Blazquez et al., 2005) and a homozygous duplication mutation in exon 8 of the *TYMP* gene (Gamez et al., 2005). The majority of these mutations are loss of function mutations. Heterozygous mutation carriers are asymptomatic with approximately 35% residual TP activity, although the plasma nucleoside levels are similar to healthy controls (Marti et al., 2004).

In addition, non-pathogenic polymorphisms have been described in the *TYMP* gene. The A465T polymorphism (c.1393G>A) was reported both in subjects with MNGIE like features and control subjects (Vissing et al., 2002; Martin et al., 2004). In some MNGIE cases there is no or mild clinical involvement of gastrointestinal tract or skeletal muscle, despite the presence of mutations in the *TYMP* gene leading to marked reduction in TP activity, probably indicating that environmental factors contribute to the severity of the clinical symptoms (Martin et al., 2004; Szigeti et al., 2004b). Apart from late-onset forms of the disease (Marti et al., 2005; Massa et al., 2009; Etienne et al., 2012), most patients display typical MNGIE features before the age of 20 years (Nishino et al., 2000; Teitelbaum et al., 2002).

### Clinical Manifestations

Gastrointestinal and ocular involvements are usually the first complications in this disease, although neuropathy and hearing loss have been reported as primary symptoms in some cases (Garone et al., 2011). Clinical symptoms are summarized in **Table 1**.

**Table 1:** Common and rare clinical symptoms in MNGIE patients.

Complication	Symptoms	Pathophysiology	Remarks	Study
Gastrointestinal	Appetite loss, satiety	- Myogenic (visceral smooth muscle): atrophy in the muscularis propria of the stomach and small intestines	-A major cause of death and survival is generally related to the severity of these symptoms	Perez-Atayde et al., 1998; Blondon et al., 2005; Giordano et al., 2008; Zimmer et al., 2009; Granero Castro et al., 2010; Garone et al., 2011; Chapman et al., 2014
	Weight loss	- Neurogenic (enteric nervous system): loss of the interstitial cells of Cajal	- Can lead to severe denutrition, anemia and eventually the necessity for nutritional supportive treatments	
	Digestive features: chronic diarrhea, abdominal pain, cramps, nausea, colonic distension, dysphagia	-Mixed myo-neurogenic causes	- CIPO in the early disease course is under recognized	
Ocular	External ophthalmoplegia, ptosis, retinal pigmentary changes, glaucoma, optic nerve atrophy		CPEO phenotype is often present. Recovered upon HSCT transplantation compared to untreated patient	Threlkeld et al., 1992; Barboni et al., 2004; Vinciguerra et al., 2015
Auditory	Deafness	-Dysfunction of cranial nerve and auditory cortex - Atrophy of the stria vascularis in the cochlea	- Hearing loss is common among patients (in 61% of patients)	Hirano et al., 1994; Yasumura et al., 2003; Li et al., 2011; Mattman et al., 2011
			- Satisfactory results were obtained soon following cochlear implantation in MNGIE patients	
CNS	Mental changes, subcortical loss of cognitive functions, memory impairment	leukencephalopathy	- MNGIE is an example of an adult mitochondrial disorder in which leukodystrophy is observed	Millar et al., 2004; Barragan-Campos et al., 2005; Scaglia et al., 2005; Carod-Artal et al., 2007; Schupbach et al., 2007; Schiffmann and van der Knaap, 2009; Salsano et al., 2013; Scarpelli et al., 2013
			- Patients presenting the characteristic multisystem symptoms of MNGIE have a unique pattern on brain MRI indicative of vasogenic oedema and glial cell dysfunction	
			- To date, it is debatable whether or not the extent of these brain MRI signal alterations, correlates with age, clinical severity, CNS involvement, or the biochemical and genetic profiles of MNGIE patients	
PNPs	Numbness and paraesthesia	Demyelinating sensorimotor type: reduced sensory motor conduction, loss of myelin sheaths in lumbar and brachial plexus	- Neuropathy usually is not among the first symptoms of the disease	Simon et al., 1990; Hirano et al., 1994; Bedlack et al., 2004; Menezes and Ouvrier, 2012; Pupe et al., 2012
			- Some MNGIE cases are misdiagnosed with chronic inflammatory demyelinating polyneuropathy	
Skeletal muscle	Proximal myopathy	mtDNA molecular alterations and abnormal respiratory chain enzymes in skeletal muscles	Two cases with classical clinical presentation of MNGIE, were reported without skeletal muscle involvement. Both cases showed identical homozygous splice-acceptor site mutation in *TYMP* gene (c.215-1G>C), which may suggest a genotype-phenotype correlation	Papadimitriou et al., 1998; Hirano et al., 2004; Szigeti et al., 2004b; Cardaioli et al., 2010; Bax et al., 2013
Others	Endocarditis		-Rare complications	Hirano et al., 1994; Yolcu et al., 2014; Kalkan et al., 2015
	Spontaneous abdominal esophageal perforation		- Short stature as seen in many mitochondrial diseases and partly as a complication of failure to thrive	
	Short stature			
	Cardiomyopathy			
	Psoriasis			


*CIPO, chronic intestinal pseudo obstruction; CPEO, chronic progressive external ophthalmoplegia; CNS, central nervous system; PNPs, polyneuropathies.*

### Diagnosis

**FIGURE 1:**
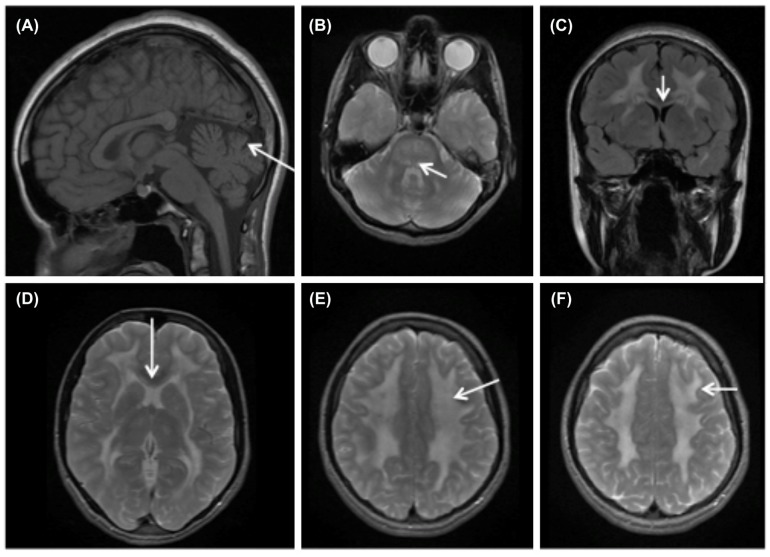
**Brain MRI findings in MNGIE.** MRI of MNGIE patient at age 16 with “typical” MNGIE phenotype. **(A)** T1 weighted sagital image shows cerebellar vermis atrophy (arrow) and normal gyral pattern. **(B)** Axial T2 with hyperintensities in the dorsal pons and mesencephalon (arrow). (**C** coronal flair image, **D** axial T2) Show extensive signal abnormalities in the cerebral white matter. The external capsule is involved as is the inner blade of the corpus callosum (arrow **C**,**D**). **(E**,**F)** Extensive white matter involvement with sparing of the U-fibers (arrow).

Detailed patient history, thorough clinical examination, particular findings on magnetic resonance imaging (MRI) of the brain (**Figure 1**), genomic DNA screening for mutations in *TYMP* gene and biochemical analysis all contribute to the diagnosis of MNGIE. Biochemical diagnosis of MNGIE includes at least one of the following parameters (Marti et al., 2004): (1) Increased blood plasma levels of dThd and dUrd (>3 and >5 μmol/L, respectively). (2) Severely reduced TP enzyme activity in buffy coat leukocytes (<8% of healthy controls; healthy control mean TP activity equivalent to 634 nmol thymine formed/hr/mg protein). Biochemical analysis reduces the risk of missing the diagnosis in case of non-identified mutation sites (Nishino et al., 2000) or in case of unclassified variants (UV). Additionally, biochemical diagnosis contributes to the confirmation or exclusion of the role of a UV as a cause for MNGIE. Similarly, biochemical assessment is preferred over clinical diagnosis since some of the classical symptoms of MNGIE can be absent. Other frequently observed findings in MNGIE patients include metabolic abnormalities such as lactic acidosis, deficiency of mitochondrial respiratory chain enzymes, mainly complex I and IV (Hirano et al., 1994; Debouverie et al., 1997), urinary Thd and dUrd accumulation (Fairbanks et al., 2002; Spinazzola et al., 2002; la Marca et al., 2006) and elevated protein levels in CSF (Bedlack et al., 2004). Infrequently, skeletal muscle biopsies may reveal ragged red fibers, and mtDNA analysis may reveal acquired deletions, depletions or point mutations (Teitelbaum et al., 2002; Nishigaki et al., 2003).

## Pathogenesis

The TP enzyme converts mitochondrial dThd and dUrd to the nucleotide bases thymine and uridine respectively and 2-deoxy ribose 1-phosphate (Friedkin and Roberts, 1954). This occurs in *de novo* synthesis or via the salvage pathway. dThd and dUrd are homogeneously present in cellular and plasma compartments and they translocate between compartments through NTs. In humans two unrelated protein families have been described (Young et al., 2013), CNTs, an active transport system, and ENTs responsible for passive facilitated diffusion.

**FIGURE 2:**
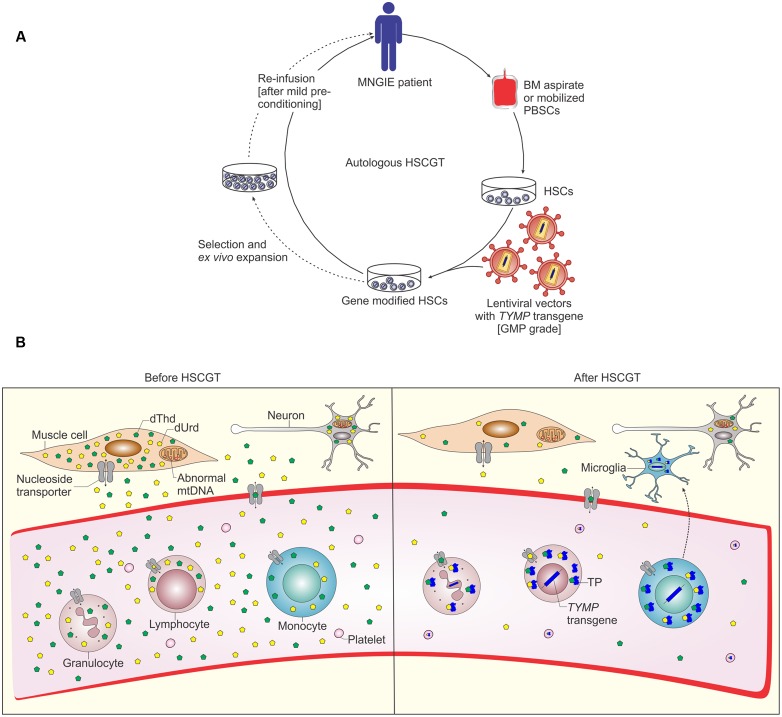
**Schematic representation of autologous hematopoietic stem cell based gene therapy for MNGIE and possible mechanism of biochemical correction by gene modified HSCs.** **(A)** Autologous bone marrow (BM) aspirates or apheresis of peripheral blood HSCs (PBSCs) after treatment with rh-G-CSF or plerixafor are collected from MNGIE patient. HSCs are *ex vivo* transduced by GMP grade lentiviral vectors containing the human *TYMP* transgene. Before infusion of the transduced cells, MNGIE patients are pre-treated with non-myeloablative conditioning to allow minimal engraftment of gene modified HSCs. Selection and *ex vivo* expansion of gene modified HSCs allows for transplantation of large numbers of gene modified HSCs to obviate the need for myeloablative pre-conditioning and allows (to some degree) for assessment of safety of the gene modified HSCs prior to transplantation, for example by lentiviral vector integration analysis (reviewed in Watts et al., 2011). **(B)** The enzyme thymidine phosphorylase (TP) is deficient in all tissues of MNGIE patients, which leads to accumulation of the nucleoside substrates dThd and dUrd and depletion of the nucleotide dCTP and finally mtDNA depletion and deletion (Gonzalez-Vioque et al., 2011). Following transplantation of gene modified HSCs and homing to bone marrow, these cells differentiate into all types of blood cells, LV genome and human *TYMP* transgene are integrated in leukocyte DNA ensuring stable expression of TP. TP catalyzes the chemical reaction which breaks down the nucleosides. This process eventually leads to reduction of systemic nucleosides accumulation. NTs mediate nucleosides transfer via passive facilitated diffusion (ENTs) and active transport (CNTs), the ubiquitous bidirectional ENTs are depicted (Young et al., 2013). In addition, some gene modified HSCs differentiate into monocytes and may migrate to the brain giving rise to microglia which act as a TP reservoir and cross correct the other cells in CNS.

The bidirectional ENTs, mainly ENT1, are ubiquitously present on almost all cell types and mediate the uptake and efflux of nucleosides (**Figure 2B**). Therefore, they are important for cells that rely on the salvage pathway for supply of nucleosides, including bone marrow cells, erythrocytes and leukocytes, brain and muscles (Young et al., 2008). Although TP is not expressed in all tissues, the TP expressed in circulating platelets and leukocytes and some other tissues is essential to degrade the excess amounts of dThd and dUrd nucleosides which are secreted into the blood (Lara et al., 2007).

The molecular pathological mechanism in MNGIE involves imbalanced nucleosides and nucleotide pools. Initially, loss of function mutations in *TYMP* gene were identified resulting in reduced TP activity (Nishino et al., 1999) leading to accumulation of access amounts of the nucleoside substrates in blood plasma, urine and almost all tissues (Spinazzola et al., 2002; Valentino et al., 2007). It has been hypothesized that this biochemical imbalance disturbs the equilibrium of intra-mitochondrial dNTPs pools (Spinazzola et al., 2002) and hence is responsible for mtDNA depletion, multiple deletions, and point mutations associated with MNGIE (Hirano et al., 1994; Papadimitriou et al., 1998; Nishino et al., 2000; Nishigaki et al., 2003). Therefore, recent studies have addressed the relationship between biochemical and dNTP pool imbalances and subsequent mtDNA abnormalities in MNGIE. *In vitro*, mtDNA point mutations and deletions, similar to those detected in MNGIE patients were reported in cultured HeLa cells after long time culture in the presence of high levels of thymidine in the culture medium. These mtDNA alterations were attributed to expanded levels of dTTP and dGTP and reduced levels of dCTP and dATP. However, no mtDNA depletion was observed in these HeLa cells (Song et al., 2003). Further investigation revealed that this increase in dTTP, under similar culture conditions, was more pronounced in non-cycling skin and lung fibroblasts leading to depletion in mtDNA in a dThd dose and time dependent manner (Pontarin et al., 2006). Interestingly, mtDNA levels were recovered upon removal of the dThd from the culture medium. In order to understand the influence of metabolites accumulation on the creation of mtDNA alterations, an *in organello* experimental model was used. Excess amounts of dThd were responsible for the significant increase in mitochondrial levels of dTTP, together leading to secondary TK2 inhibition mediated reduction of dCTP nucleotides (Gonzalez-Vioque et al., 2011). Subsequent studies confirmed these findings in *in vitro* fibroblast cultures and *in vivo* in the *Tymp^-/-^Upp1^-/-^* mouse model and suggest that the inadequate availability of dCTP accounts for the mtDNA depletion observed in MNGIE (Gonzalez-Vioque et al., 2011; Camara et al., 2014; Torres-Torronteras et al., 2014).

Altogether, these studies demonstrate that indeed it is the nucleoside accumulation and subsequent reduction of dCTP nucleotides, rather than the deficiency of TP *per se*, that accounts for the molecular and phenotypic alterations in MNGIE. An excellent illustration of this observation is the fact that TP expression in skeletal muscles is absent, nonetheless, some but not all MNGIE cases were reported with skeletal muscle mtDNA deletions, histological and oxidative phosphorylation abnormalities (Papadimitriou et al., 1998; Hirano et al., 2004).

When available, although limited, analysis of *postmortem* MNGIE samples is relevant and beneficial to gain knowledge about the molecular and pathological basis of the disease. Severe intestinal dysmotility, also known as CIPO, and weight loss are principle presentations of MNGIE. Histopathological analysis of MNGIE gastrointestinal samples revealed depletion in mtDNA and mitochondrial proliferation, and consequently cell atrophy in the muscularis propria layer of the stomach and small intestines (Giordano et al., 2006, 2008). Additionally, loss of interstitial cells of Cajal and morphologically abnormal muscularis propria and ganglion cells have been reported (Zimmer et al., 2009). On the other hand, the study of brain tissues of two MNGIE patients revealed no pathological proliferation of glial cells nor neuronal loss. However, the study suggested a role of TP deficiency in impairment of BBB, which could contribute to the observed hyperintense T2 signals on brain MRI scans (Szigeti et al., 2004a).

Nucleoside accumulation is detrimental probably during the early course of the disease, because nucleoside clearance did not improve mtDNA content per cell or reduce COX deficient fibers after liver transplantation (De Giorgio et al., 2016). Mitochondrial DNA instability is a hallmark for diseases caused by defective nuclear genes essential for mtDNA replication and repair (such as *PEO1, POLG1,2*) or maintenance of dNTP pools (such as *ANT1, TYMP*) or others involved in mtDNA homeostasis (such as FBXL4) (Young and Copeland, 2016). Mutations in PEO1, POLG and ANT1 underlie the autosomal dominant form of progressive external ophthalmoplegia (adPEO), a very well-characterized mtDNA disorder involving stalling of mtDNA replication (Van Goethem et al., 2001; Goffart et al., 2009). Therefore, stalling of Twinkle helicase or DNA polymerase γ could be a common pathological mechanism underlying mtDNA instability in MNGIE, PEO and mtDNA depletion syndrome (Hirano et al., 2001; Liu et al., 2008).

## Current Treatments For MNGIE

In general, treatment of mitochondrial diseases is mainly based on symptom management and supportive care (Pfeffer et al., 2013). Vitamin and amino acid supplements (Tanaka et al., 1997) and exercise therapy (Taivassalo et al., 1998) aiming to improve mitochondrial functions are recommended for mitochondrial myopathies. Symptomatic management of MNGIE consists of nutritional support (Wang et al., 2015), prevention of infections and pain relief including interventions such as celiac plexus neurolysis and blockage of the splenic nerve (Teitelbaum et al., 2002; Celebi et al., 2006). Since the metabolic and mtDNA abnormalities are attributed to the systemic nucleoside imbalances, clinical interventions focus on direct removal of these metabolites to restore the balance or by introducing the deficient enzyme to reduce the metabolites.

### Hemodialysis and Peritoneal Dialysis

The first hemodialysis aiming to remove the excess amounts of nucleosides from the circulation was performed in Spinazzola et al. (2002) in two MNGIE patients followed by another in 2006. In the first two patients, significantly reduced thymidine levels were observed shortly after hemodialysis, however this effect was transient as thymidine levels returned to pre-dialysis levels 3 h after dialysis (Spinazzola et al., 2002). A progressive reduction in Thd levels below the basal levels was observed after repetitive dialysis treatments in the third case (la Marca et al., 2006). A MNGIE patient who had peritoneal dialysis showed an improvement in gastrointestinal symptoms (such as vomiting, anorexia, abdominal pain, and diarrhea) during the continuing peritoneal dialysis for 3 years with body weight gain, although other major symptoms including ocular and neurological abnormalities and brain MRI signals did not change (Yavuz et al., 2007). Another case report noted improvement of the gastrointestinal and neurological symptoms, mainly the mitigation of numbness in the hands, until nucleoside levels increased again 15 months after continuous ambulatory peritoneal dialysis (Ariaudo et al., 2014).

### Enzyme Replacement Therapy

Initially, platelet infusions were performed in two MNGIE patients to restore TP enzyme activity in the blood. This approach showed efficient recovery of functional TP enzyme and correction in nucleoside imbalances, however, like dialyses, these improvements were temporary requiring multiple treatment sessions for long-term responses. ERT is a reliable, well-tolerated approach to replace the deficient enzyme in a variety of lysosomal storage disorders including Gaucher, Pompe and Fabry disease, and Sly syndrome (Wilcox et al., 2004; Burrow et al., 2007). For MNGIE, approaches were developed to encapsulate TP in order to prolong the half-life of circulatory TP enzyme and reduce the immunogenic reactions. These include polymeric nanoparticles (De Vocht et al., 2009) and erythrocytes as they are permeable and affect the plasma metabolites, such as in adenosine deaminase deficiency (Bax et al., 2000; Moran et al., 2008). The EE-TP concept is under clinical development as an orphan ERT for MNGIE with the first attempt carried out in a MNGIE patient in Moran et al. (2008). In this approach, autologous erythrocytes were isolated from patients and loaded with recombinant *Escherichia coli* TP enzyme *in vitro* via hypo osmotic dialysis. Significant clinical improvements were observed such as the ability to walk and climb and the recovery of sensation and the mitigation of numbness in hands and feet, even after 23 months after termination of multiple cycles of EE-TP (Bax et al., 2013). When using the EE-TP approach, there is a high risk that an immunological reaction is triggered against the bacterial TP, especially if the infusions are repeated several times, although this has not been observed (Levene et al., 2013).

### Orthotopic Liver Transplantation

Liver transplantation is a new ERT strategy for treatment of MNGIE patients. TP protein levels are high in healthy human liver tissues and significantly higher than in bone marrow cells (Boschetti et al., 2014). Recently, OLT was successfully applied in a severely affected, 25-years old MNGIE patient (De Giorgio et al., 2016). Steady state nucleoside balance was observed up to13 months post OLT. Slight improvements in lower limb strength and brain metabolism (reduced lactate levels) and structure (reduced cerebellar mean diffusivity values at diffusion MRI), improved quality of life scores and nutritional parameters, but not body weight (40 kg), were observed up to 6 months after OLT. When ileostomy closure was performed, the gastrointestinal (GI) functions and body weight declined at 13 months (37 kg). Therefore, it remains uncertain whether the mild restoration of GI function was due to the decompressive ileostomy, instead of the OLT. In addition, skeletal muscle mtDNA content per cell was slightly increased after OLT. The study suggests that the damage in post-mitotic tissues during late stages of the disease is irreversible despite recovery of nucleoside balance. Therefore, biochemical correction should probably be achieved prior to irreversible damage, preferably before the intestinal symptoms appear. Preoperative conditioning for OLT is not required. However, this approach requires matched organ donors (which are limited), involves transplantation related risks and requires long-term immunosuppression which all can further affect the quality of life of the patients.

### Hematopoietic Stem Cell Transplantation

Another possibility to restore TP enzyme activity in the circulation is by HSCT. Recently, a retrospective analysis of all HSC transplanted MNGIE patients between 2005 and 2011 showed that only nine out of 24 patients were alive up to 4 years after transplantation. All nine survivors had normalized TP activity in their blood while seven of them showed improved body mass index, gastrointestinal symptoms and peripheral neuropathy. On the other hand, nine MNGIE patients died mainly due to transplant-related causes such as GVHD and graft failure, including recipients of HLA-mismatched unrelated cord blood transplants, while the remaining six patients died of disease progression. The recommendations of this study included transplantation of a sufficient number of cells, because in some patients the graft was rejected, and to consider more closely HLA matched donor cells, because of the large number of GVHD observed in this retrospective study, and to transplant at an earlier age before major organ damage has occurred (Halter et al., 2011, 2015).

The poor physical state of MNGIE patients when they enroll HSCT trials increases the risk for transplantation related complications caused by conditioning regimens and immune-suppressants. Other problems may arise from the drugs used in HSCT which are potentially harmful to mitochondria such as cyclophosphamide (Mariana Ponte Cardoso et al., 2015). Therefore, MNGIE patients are treated with Busulfan and fludarabine prior to HSCT, following the recommendations of MNGIE consensus meeting in Halter et al. (2011). For MNGIE patients who develop liver cirrhosis, allogeneic HSCT (AHSCT) should be contraindicated and OLT would be the treatment of choice (Finkenstedt et al., 2013). Pre-existing liver cirrhosis complicates liver failure which may develop after AHSCT due to multiple factors such as viral infections in immunocompromised recipients or due to hepatotoxic conditioning drugs.

## Pre-Clinical Experimental Approaches Toward Therapy

### Models of MNGIE

*In organello* experiments and human MNGIE fibroblasts were used for highlighting parts of the molecular mechanism of MNGIE (Gonzalez-Vioque et al., 2011; Camara et al., 2014). To study therapeutic interventions, such as gene therapy, *in vivo* models are required. A mouse model was developed by targeted disruption of exon 4 of the *TYMP* gene to generate *Tp*^-^*^/^*^-^ mice. In contrast to human TP, murine TP degrades both dThd and dUrd; *Tp*^-^*^/^*^-^ mice were crossed with *Upp1*^-^*^/^*^-^ to generate the *Tymp*^-^*^/^*^-^*Upp1*^-^*^/^*^-^ mice, which are currently the only relevant *in vivo* animal model (Haraguchi et al., 2002; Lopez et al., 2009). *Tymp*^-^*^/^*^-^*Upp1*^-^*^/^*^-^ mice show increased levels of the purine nucleosides dThd and dUrd in plasma and tissues. Diffuse leukoencephalopathy manifests late during the lifetime of these animals, around the age of 22 months (Lopez et al., 2009). Other symptoms associated with MNGIE, such as decreased motor coordination and gastrointestinal features have not been reported in this mouse model. Brain mtDNA depletion was not consistently found in this mouse model (Lopez et al., 2009; Torres-Torronteras et al., 2011; Camara et al., 2014). Therefore, high doses of exogenous nucleosides were administered to exacerbate the mitochondrial phenotype (Garcia-Diaz et al., 2014), an approach that was rationalized by the lower nucleoside levels in *Tymp*^-^*^/^*^-^*Upp1*^-^*^/^*^-^ mice compared to MNGIE patients. Mice were on an exogenous dThd and dUrd diet for a long time (24 months) before pronounced mtDNA depletion, diffuse leukoencephalopathy and motor abnormalities were observed.

Experimental approaches have been explored for treatment of MNGIE; among which experiments performed by Camara et al. (2014) which suggest that modulation of dNTP metabolism through increasing the availability of dCTP or inhibition of its catabolism can indeed reverse and prevent, at least, dCtd imbalance. A strategy that can be applied for other similar mitochondrial disorders that are caused by altered nucleosides and dNTP metabolism, for example in disorders caused by mutations in *TK2* or *DGUOK* deficiency (Camara et al., 2014).

### Gene Therapy

The *Tymp^-/-^Upp1^-/-^* mouse model has also been used for testing potential curative treatments. A recently investigated strategy is the use of gene therapy. Both lentiviral (LV) and adeno- AAV vector mediated *TYMP* gene transfer have been evaluated in pre-clinical studies for treatment of MNGIE.

#### AAV-Mediated Liver Directed Gene Therapy

Adeno-associated viral vector gene therapy has been explored in clinical trials for a variety of inherited and acquired diseases (Naldini, 2015). The main limitation of this approach is the human immune response to AAV capsid, as demonstrated in hemophilia B trials. In one of the first AAV trials targeting the liver, therapeutic levels of FIX were achieved at a high vector dose (2 × 10^12^ vector genomes per kilogram of body weight, vg/kg). Nonetheless, this high vector dose was associated with an early decline of FIX (∼8 weeks after treatment) due to T-cell immunity against AAV capsid antigens eliminating transduced hepatocytes (Manno et al., 2006).

The hybrid vector AAV2/8 with modified molecular configuration (packaged double stranded genome) and improved cassette design (codon optimized hFIX) to enhance transduction and translational efficiency was explored in a hemophilia B trial (Nathwani et al., 2006). Stable FIX expression diminished use of the costly FIX concentrate and importantly, clinical improvement was achieved in a dose dependent manner. The least bleeding episodes were seen in recipients of the highest AAV dose (steady state 5% of normal levels at a single vector peripheral vein infusion of 2 × 10^12^ vg/kg up to 4 years) (Nathwani et al., 2014). For MNGIE, an AAV2/8 expressing human *TYMP* under the control of hepatic promoter was used for treatment of *Tymp^-/-^Upp1^-/-^* mice (Torres-Torronteras et al., 2014). Low AAV doses (2 × 10^11^ vg/kg) were sufficient to reduce nucleoside imbalances to normal levels in liver, skeletal muscle, and brain for up to 8 months, while higher doses reduced nucleosides below detection levels. However, only at higher doses (>2 × 10^11^ vg/kg) TP activity was increased in the liver (but not in skeletal muscle or brain).

In light of the clinical data of the hemophilia B trial which shows that clinical improvement was AAV dose dependent (Nathwani et al., 2014), the question for MNGIE is whether or not the low AAV dose would be sufficient to reverse a clinical phenotype beyond biochemical correction. MNGIE mouse studies failed to report any relevant clinical phenotype in *Tymp^-/-^Upp1^-/-^* mice, and therefore the potential or required dosage to cure it has not been demonstrated (Torres-Torronteras et al., 2011, 2014). Importantly, upon AAV treatment nucleosides accumulation was not reduced in the intestine of treated mice at the highest dose (10^13^ vg/kg) administered. Since the intestines are heavily affected in MNGIE patients it is important to obtain evidence of correction in this organ. Preclinical studies in hemophilic dogs and non-human primates could predict the therapeutic dose in human trials (Manno et al., 2006; Nathwani et al., 2014) if that is the case for MNGIE too, biochemical correction in the intestine might require improved expression cassettes to enhance protein production, targeting the expression to major affected organs, or the less favorable option of using higher AAV doses (>10^13^ vg/kg). High doses, for example 7.2 × 10^12^ vg/mouse were sufficient to transduce 100% of mouse hepatocytes (Nakai et al., 2005). Such high doses might be required for gene therapy of systemic diseases, i.e., when non-hepatic tissues are also affected, as in MNGIE. However, these high AAV doses are likely to cause hepatocellular toxicity, biodistribution to other unwanted organs and shedding of the AAV, enhanced risk of eliciting immunity toward the viral capsid and increased costs of virus production. Immunity against ectopic TP might be an additional concern for MNGIE patients, therefore prophylactic immunosuppression might be required. Additional pre-clinical studies have to address the possibility of an immune response against ectopic TP in previously untreated patients. In addition to increased liver TP activity correlating with vector dose, an unexpected increase in liver dGTP of *Tymp^-/-^Upp1^-/-^* mice was observed in a dose depend manner as well, although the consequences of this increase are unknown. Together these findings suggest that studies into optimal dosing of AAV may be required for clinical application.

Human AAV trials should be carried out cautiously as they can reveal complications that were not observed during preclinical studies. An example is the early decline in FIX expression (Manno et al., 2006) and hepatotoxicity observed in 4/6 recipients of a high AAV dose (2 × 10^12^ vg/kg) (Nathwani et al., 2014), due to immunity against AAV capsid. MNGIE patients are often >12 years and probably have been pre-exposed to AAV and consequently can mount strong immune responses to AAV. Therefore, individuals with neutralizing antibodies to AAV should be excluded from clinical trials to avoid an immune response toward AAV. Another concern is the durability of transgene expression considering the longer lifespan of humans, compared with the animals in preclinical studies, and the potential need for recurrent AAV injections, especially at lower vector doses. In this respect HSCGT would provide a preferable option as a single, long lasting intervention method. Additional concerns related to AAV mediated gene therapy include purity of AAV preparations and manufacturing costs (Mingozzi and High, 2013).

#### LV-Mediated Hematopoietic Stem Cell Gene Therapy (HSCGT)

The encouraging therapeutic outcomes and favorable safety profile renders LV-HSCGT an attractive therapeutic approach for a variety of hereditary metabolic disorders (Wagemaker, 2014), and is potentially advantageous over AHSCT for certain selected diseases (Naldini, 2015). Proof of concept of HSC gene therapy was obtained in *Tymp^-/-^Upp1^-/-^* mice (Torres-Torronteras et al., 2011) using a phosphoglycerate kinase promoter driving native human *TYMP* cDNA and a GFP reporter in hematopoietic cells resulting in biochemical correction in peripheral blood (Torres-Torronteras et al., 2011). More recently, we developed clinically applicable LVs that carry human *TYMP* cDNA, and demonstrated long-term biochemical correction in *Tymp^-/-^Upp1^-/-^* mice at low vector copy number (VCN). Our data demonstrates the feasibility to further develop clinical protocols for HSCGT for MNGIE (Yadak et al., 2015). Similar results in a long-term follow up of 20 months confirms the correction of biochemical imbalances which was maintained at low VCN and chimerism (Torres-Torronteras et al., 2016).

In HSCGT for MNGIE, HSCs are isolated from MNGIE patients, transduced *ex vivo* by LV vectors carrying a functional copy of *TYMP* and infused back into the patient (**Figure 2A**). The newly formed HSCs and its progenitors produce TP which catabolize the excess amounts of nucleosides (**Figure 2B**). Since the patient’s own stem cells are used, GVHD is not a concern. However, mild prophylactic immunosuppression maybe required to prevent possible immune reaction against the TP transgene.

Myeloablative pre-conditioning might be necessary for high levels of engraftment in other metabolic disorders, such as metachromatic leukodystrophy (MLD), due to lack of selective advantage of gene modified cells. In particular, busulfan myeloablative conditioning is used in MLD patients for depletion of endogenous microglia and mobility of gene modified monocytes through the BBB (Capotondo et al., 2012). The contribution of gene modified microglia to correct biochemical imbalances has never been explored in MNGIE. However, murine gene therapy studies using LV and AAV vectors implicate reversal of nucleoside imbalance at low or possibly no increase in brain TP activity (Torres-Torronteras et al., 2014, 2016). In liver directed AAV2/8 gene therapy, it is not expected that brain cells will be transduced. In HSCGT, gene modified monocytes are expected to migrate to brain and differentiate into microglia. Nonetheless, the results of the HSCGT MNGIE mouse study do not rule out the potential that gene modified microglia can contribute to correction of brain biochemistry and phenotype, although this might not be necessary if ectopic expression outside the brain is high enough. When transduction efficiency is high enough, significant TP activity can be measured in the brain, indicating that transduced microglia might reside in the brain after long-term follow-up. To that end, two potential mechanisms might act synergistically to normalize the brain nucleoside levels, a systemic ectopic source and one local contribution of gene-modified cells (**Figure 2B**).

Potential options and future research for application in MNGIE patients include alternative conditioning strategies to obviate the cytotoxicity related to myeloablative conditioning and strategies to enhance the quality of infused gene modified HSCs. One approach is to mobilize endogenous HSCs into peripheral blood in order to create (space) in the bone marrow for the infused donor HSCs to engraft (Chen et al., 2006). Human G-CSF was sufficient in immunocompromised mice (Huston et al., 2014), probably due to the selective advantage of the gene modified cells, however, more stringent agents might be required in normal immunocompetent mice. A possibility is G-CSF in combination with the more potent HSCs mobilizer plerixafor (a specific CXCR4 antagonist) or the selectins inhibitor fucoidan. Such regimens probably require additional mild chemotherapeutics, in particular if the gene corrected TP-expressing HSCs lack selective growth advantage to overcome host cells. These HSCs mobilizers act via different mechanisms, therefore parameters such as the optimal dose and time frame for transplantation after mobilization need to be established in relevant pre-clinical models. Alternatively, targeting specific endogenous hematopoietic populations might reduce the off-target toxicity related to the common non-specific conditioning (Aiuti and Naldini, 2016). Examples include inhibiting c-kit, a HSC tyrosine kinase cell surface antigen (Xue et al., 2010) and the recently developed immunotoxin against hematopoietic stem cells (CD45-SAP) (Palchaudhuri et al., 2016).

Strategies such as *ex vivo* expansion of gene modified HSCs can improve the quality of the infused gene modified cells and enhance the outcome of gene therapy (Watts et al., 2011). In particular when combined with additional approaches to enrich for HSCs, preserve stemness of- and enhance homing and engraftment ability of gene modified HSCs (Psatha et al., 2016). Ultimately, this approach combined with improved mild pre-conditioning protocols, could benefit patients in poor health condition at transplantation, such as in MNGIE patients.

A risk of HSC gene therapy is insertional mutagenesis. The first HSC gene therapy trials used gammaretrovirus (γ-RV) based vectors for treatment of SCID-X1 (Gaspar et al., 2004; Hacein-Bey-Abina et al., 2010), adenosine deaminase (ADA-SCID) (Aiuti et al., 2002), CGD (Ott et al., 2006) and WAS (Boztug et al., 2010). Although efficient correction of immunodeficiency was achieved in most patients in SCID-X1, CGD and WAS trials, lympho-proliferative disorders (Hacein-Bey-Abina et al., 2003a,b, 2008; Howe et al., 2008; Braun et al., 2014) and myelodysplasia (Stein et al., 2010) developed secondary to γ-RV vector integrations within or nearby proto-oncogenes. In addition to the preferred integration profiles over γ-RV vector (Deichmann et al., 2007; Cattoglio et al., 2007; Gabriel et al., 2012), LV efficiently transduce non-cycling primitive HSCs and under minimum culture conditions (Naldini et al., 1996; Guenechea et al., 2000). Therefore, attention was focused on development of LV as a relatively safer approach, leading eventually to development of third-generation SIN-LV (Dull et al., 1998; Zufferey et al., 1998). Several pre-clinical studies indicate the reduced genotoxicity of SIN-LV vectors compared with γ-retroviral vectors, in particular SIN-LV CISs revealed no preference of integration near proto-oncogenes (Montini et al., 2006; Modlich et al., 2009; Biffi et al., 2011; Romero et al., 2013; Zhou et al., 2013). Since then, SIN-LV vectors have been applied successfully in ongoing clinical trials for a variety of metabolic (Cartier et al., 2009; Biffi et al., 2013) and immunodeficiency disorders (Aiuti et al., 2013), and no adverse events have yet been reported in these trials. Moreover, the therapeutic benefits without toxicity related to transgene expression and biosafety of SIN-LV vectors has been further validated through a growing body of recent preclinical studies supporting the initiation of clinical trials, for example for β-thalassemia (Negre et al., 2015) and mucopolysaccharidosis I disease (Visigalli et al., 2016).

Furthermore, selective advantage for growth and differentiation conferred by the therapeutic transgene expression increases the potential risk for proliferative disorders, this was reported in some immunodeficiency conditions (Aiuti and Roncarolo, 2009). For metabolic disorders, for instance lysosomal storage disorders, however, most studies show that enzyme positive cells have no selective advantage (Bernardo and Aiuti, 2016), which is most likely the case in MNGIE as well. To improve safety, technologies such as *ex vivo* expansion of gene modified HSCs may permit for safety assessment (to some degree) prior to transplantation, by analysis of LV integration sites (Watts et al., 2011; **Figure 2A**).

#### AAV Mediated GT or HSCGT?

Regardless of the type of viral vector used for gene therapy, the chosen strategy should provide long-term expression of the gene of interest without side effects in the host. It is important to apply a well-defined vector dose that is sufficient to reverse the biochemical and nucleotide imbalance without any potential side effects. In particular, abnormal overexpression of the TP enzyme is detected in different tumor types, including non-small cell lung-, colorectal-, breast-, gastrointestinal-, and hepatic cancers (Koukourakis et al., 1997; Mori et al., 2000; Ikeguchi et al., 2001; Nakayama et al., 2005; Mitselou et al., 2012) and correlates with a worse prognosis in colorectal cancer patients (Takebayashi et al., 1996). Besides, disturbance of dNTP pools can be a trigger for cell cycle arrest and apoptosis (Oliver et al., 1996; Kumar et al., 2010).

The medical condition of the patient can also influence the choice of the vector system for clinical application. For terminally ill patients, the AAV approach could be most suitable to avoid the risks associated with the pre-conditioning for transplantation in autologous HSC gene therapy or if a suitable HSCs donor for AHSCT is lacking.

## Concluding Remarks

The lack of mitochondrial histone protection, the limited repair capacity and oxidized dNTPs contributing to mismatch errors (Alexeyev et al., 2013) all make mitochondria more susceptible than nuclear DNA to mutagenesis. It has become evident that it is the systemic accumulation of nucleosides in MNGIE (Di Meo et al., 2015) that causes imbalances in mitochondrial dNTP pools. However, the mechanism by which it causes mtDNA alterations is still unknown. Although the current treatments focus on restoration of TP enzyme activity and/ or elimination of accumulating metabolites, further understanding of cellular mechanisms involved in maintenance of mtDNA integrity and copy number can provide targets for clinical intervention for MNGIE and possibly other mitochondrial disorders.

Platelet infusions, hemato/peritoneal dialysis, and EE-TP ERT could be used to provide biochemical correction. AAV gene therapy and lentiviral HSCGT are potential curative options as evidenced by the promising pre-clinical results in *Tymp^-/-^Upp1^-/-^* mice. OLT is a promising emerging treatment and should currently be the treatment of choice for MNGIE patients with pre-existing liver failure. Allogeneic HSCT has risks of graft failure, GVHD and conditioning-related toxicity. Milder conditioning may be applicable in HSCGT, and treatment should preferably be applied at an early age. Novel strategies are being explored to improve the safety and efficiency of viral based gene therapy, ultimately for MNGIE patients as well. These include strategies to enhance transduction, improve engraftment of gene modified HSCs and limit transplantation related toxicity, and others to overcome the limitation of AAV capsid triggered immunity by means of novel serotypes and improved transcription cassettes.

Mitochondrial neurogastrointestinal encephalomyopathy patients should receive suitable treatment promptly before permanent damage occurs, which can be challenging, as MNGIE patients are often diagnosed late during disease progression in a poor health condition. Because TP activity and nucleoside levels can be routinely measured in blood samples, MNGIE should be considered to be included in newborn screening programs, similar to other (neuro) metabolic disorders for early diagnosis and treatment (Carlson, 2004; McHugh et al., 2011).

## Author Contributions

RY framed the structure of the review, analyzed the literature and wrote the manuscript; PS, MvG participated in the literature analysis; NvT, IdC participated in the literature analysis and supervised the writing. All authors discussed the topic and provided intellectual feedback to the article. All the authors read and approved the manuscript.

## Conflict of Interest Statement

The authors declare that the research was conducted in the absence of any commercial or financial relationships that could be construed as a potential conflict of interest.

## Footnotes

1 http://www.orpha.net/consor/cgi-bin/index.php

## Abbreviations

AAV: adeno-associated virus
ADA-SCID: adenosine deaminase-severe combined immunodeficiency
AHSCT: allogeneic hematopoietic stem cell transplantation
ANT-1: adenine nucleotide translocase type 1
BBB: blood–brain barrier
CGD: chronic granulomatous disease
CIPO: chronic intestinal pseudo obstruction
CIS: common integration site
CNT: concentrative nucleoside transporter
CPEO: chronic progressive external ophthalmoplegia
dATP: deoxyadenosine triphosphate
dCTP: deoxycytidinetriphosphate
dGTP: deoxyguanosine triphosphate
DGUOK: deoxyguanosine kinase
dNTP: deoxyribonucleoside triphosphates
dThd: thymidine
dTTP: deoxythymidine triphosphate
dUrd: deoxyuridine
ECGF1: platelet-derived endothelial cell growth factor
EE-TP: erythrocyte encapsulated TP
ENT: equilibrative nucleosides transport
ERT: enzyme replacement therapy
FBXL4: F-box and leucine-rich repeat protein 4
FIX: coagulation factor IX
G-CSF: granulocyte colony stimulating factor
GVHD: graft versus host disease
HSCT: hematopoietic stem cell transplantation
HSCGT: hematopoietic stem cell gene therapy
LHON: Leber’s hereditary optic neuropathy
LV: lentivirus
MDS: mitochondrial DNA depletion syndrome
MELAS: mitochondrial encephalomyopathy, lactic acidosis, and stroke-like episodes
MNGIE: mitochondrial neurogastrointestinal encephalomyopathy
NT: nucleoside transporter
OLT: orthotopic liver transplantation
PEO: progressive external ophthalmoplegia
POLG: DNA polymerase subunit gamma
RRM2B: ribonucleotide reductase M2 B
SCID- X1: X-linked severe combined immunodeficiency
SIN-LV: self-inactivating LV
TK2: thymidine kinase 2
TYMP: thymidine phosphorylase
WAS: Wiskott–Aldrich syndrome
γ-RV: gammaretrovirus
